# Middle east respiratory syndrome corona virus spike glycoprotein suppresses macrophage responses *via* DPP4-mediated induction of IRAK-M and PPARγ

**DOI:** 10.18632/oncotarget.14754

**Published:** 2017-01-19

**Authors:** Ahmed A. Al-Qahtani, Konstantina Lyroni, Marina Aznaourova, Melpomeni Tseliou, Mashael R. Al-Anazi, Mohammed N. Al-Ahdal, Saad Alkahtani, George Sourvinos, Christos Tsatsanis

**Affiliations:** ^1^ Department of Infection and Immunity, Research Center, King Faisal Specialist Hospital and Research Center, Saudi Arabia; ^2^ Department of Microbiology and Immunology, School of Medicine, Alfaisal University, Riyadh, Saudi Arabia; ^3^ Laboratory of Clinical Chemistry, Medical School, University of Crete, Heraklion, Greece; ^4^ Laboratory of Virology, Medical School, University of Crete, Heraklion, Greece; ^5^ Zoology Department, College of Science, King Saud University, Riyadh, Saudi Arabia

**Keywords:** MERS CoV, macrophages, DPP4, IRAK-M, cytokines, Immunology and Microbiology Section, Immune response, Immunity

## Abstract

Middle East Respiratory Syndrome Corona Virus (MERS-CoV) is transmitted *via* the respiratory tract and causes severe Acute Respiratory Distress Syndrome by infecting lung epithelial cells and macrophages. Macrophages can readily recognize the virus and eliminate it. MERS-CoV infects cells *via* its Spike (S) glycoprotein that binds on Dipeptidyl-Peptidase 4 (DPP4) receptor present on macrophages. Whether this Spike/DPP4 association affects macrophage responses remains unknown. Herein we demonstrated that infection of macrophages with lentiviral particles pseudotyped with MERS-CoV S glycoprotein results in suppression of macrophage responses since it reduced the capacity of macrophages to produce TNFa and IL-6 in naive and LPS-activated THP-1 macrophages and augmented LPS-induced production of the immunosuppressive cytokine IL-10. MERS-CoV S glycoprotein induced the expression of the negative regulator of TLR signaling IRAK-M as well as of the transcriptional repressor PPARγ. Inhibition of DPP4 by its inhibitor sitagliptin or siRNA abrogated the effects of MERS-CoV S glycoprotein on IRAK-M, PPARγ and IL-10, confirming that its immunosuppressive effects were mediated by DPP4 receptor. The effect was observed both in THP-1 macrophages and human primary peripheral blood monocytes. These findings support a DPP4-mediated suppressive action of MERS-CoV in macrophages and suggest a potential target for effective elimination of its pathogenicity.

## INTRODUCTION

The Middle East respiratory syndrome Corona virus (MERS-CoV) is a positive-sense, single-stranded RNA beta-coronavirus, related to the severe acute respiratory syndrome coronavirus (SARS-CoV). MERS-CoV was identified in humans for the first time in 2012, in the Kingdom of Saudi Arabia [1] and as of June 2015, more than 1,330 cases and 475 associated deaths had been confirmed (http://www.who.int/csr/disease/coronavirus_infections/archive_updates/en) [2]. A single exported case with travel history in the Middle East has resulted in an ongoing outbreak in the Republic of Korea, while MERS-CoV cases have also been detected in Europe, USA, Asia and Africa. Clinical features of MERS-CoV infection vary from asymptomatic cases to severe lower respiratory tract infection, shock, multiorgan failure which may lead to death [3, 4]. The virus can spread from person-to-person [5] although the rate of transmission appears to be low [6, 7], probably due to the low percentage of respiratory epithelial cells which are truly infected by MERS-CoV and the increased number of viral particles which are likely needed to be inhaled to cause infection [8].

Among the major structural components [1, 9], the spike (S) glycoprotein expressed by MERS-CoV (reviewed in [10]) plays an important role in both viral attachment and entry into the target cell [11]. MERS-CoV S glycoprotein contains 1,353 amino acids and appears as a trimer on the viral membrane surface. The precursor S protein can be cleaved into two subunits, S1 and S2. The S1 subunit contains the receptor-binding domain (RBD) and is responsible for binding to the cellular receptor Dipeptidyl-Peptidase 4 (DPP4) while the subunit S2 consists of a putative fusion peptide, transmembrane domain and two hep-tad repeat regions, termed heptad repeats 1 and 2 and mediates membrane fusion [11–14]. MERS-CoV infection is mediated by the binding of viral S glycoprotein to its cognate receptor dipeptidyl peptidase 4 (DPP4/CD26) [11, 15]. DPP4 is a type-II transmembrane glycoprotein which is widely expressed on non ciliated bronchial epithelium and in macrophages [12, 16]. Determinants of host range and cellular tropism are located in the RBD region of the S glycoprotein while crystal structure modelling have revealed the amino acid residues in DPP4 receptor which are critically implicated in the S glycoprotein-DPP4 binding [12, 17, 18]. DPP4 receptor is present on different cell types that are infected by the virus, including macrophages. Whether interaction of the S glycoprotein with DPP4 alters macrophage responses remains unknown.

Recent evidence has shown that MERS-CoV induces immunosuppression escaping the host's immune surveillance, partly by promoting T-cell apoptosis [19]. MERS-CoV S glycoprotein is a viral component capable of inducing immune responses. The S protein has been shown to induce potent neutralizing antibody responses, resulting in the inhibition of the infection in different of models and means of spike protein expression [20–23]. In terms of innate immunity, the immune-modulatory mechanisms induced by the MERS-CoV S glycoprotein have not been explored so far. Macrophages are infected by the virus and the virus itself is able to replicate within these cells [24].

In the present study, we investigated the impact of MERS-CoV S glycoprotein on the responsiveness of THP-1 monocytes and macrophages to TLR4 stimulation. For this purpose lentiviral particles expressing MERS S glycoprotein were constructed and its effects on LPS-induced responses were evaluated. This virus was unable to replicate and provided a unique tool to define the impact of MERS S glycoprotein and its interaction with DPP4 on THP1 cell activation. Our results showed that MERS CoV S glycoprotein suppressed the production of TNFa and IL-6 and augmented the production of IL-10. At the same time it induced the expression of the negative regulator of TLR signaling IRAK-M and the transcriptional repressor PPARγ. Treatment with the DPP4 inhibitor sitagliptin reversed the effects of MERS-CoV S glycoprotein on macrophages.

## RESULTS

### Infection of THP-1 macrophages with lentiviral particles pseudotyped with MERS S glycoprotein suppressed cytokine production

**Figure 1:**
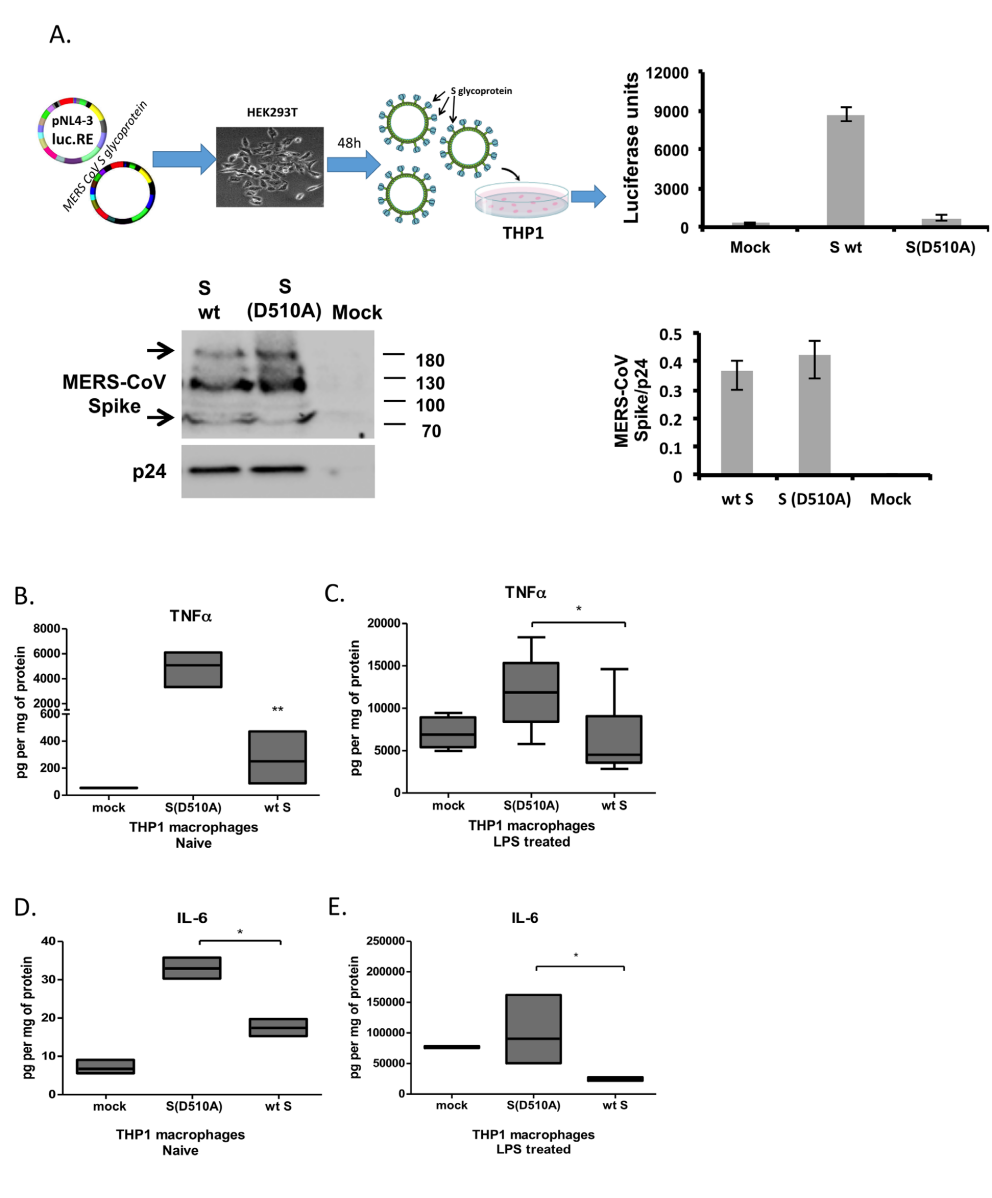
MERS-CoV S glycoprotein suppressed LPS-induced cytokine production in THP1 cells **A**. Lentiviral particles pseudotyped with MERS S glycoprotein were generated in HEK293T cells following transfection with plasmid pNL4.3R-E-luciferase and pcDNA3.1+ containing the MERS-CoV wild-type or D510A mutant spike glycoprotein. Normalized supernatants were used to infect THP-1 cells and infection was monitored by luciferase assay. The C9-tagged MERS-CoV Spike glycoprotein was detected by Western blot in purified lentiviral particles. HIV-1 p24 envelope protein was used as a normalizing control for the semi-quantitative analysis of MERS-CoV S protein among different types of pseudotyped viruses. THP-1 macrophages were infected with lentiviral particles pseudotyped with MERS wild type (wt) or the D510 mutant S glycoprotein (D510A) of MERS-CoV, or left uninfected (mock), and were stimulated with LPS. TNFα was measured in the supernatants at the naïve state **B**. and following LPS stimulation **C**. IL-6 was also measured in naïve and LPS-treated THP1 macrophages **D**., **E**. * *p* < 0.05, **p < 0.01

To determine the effect of MERS-CoV S glycoprotein on macrophage activation we generated a pseudovirus expressing the HIV backbone linked to the luciferase gene and the MERS-CoV S protein from a pCDNA3.1+ vector following transfection of the respective constructs in 293T cells. Lentiviral particles pseudotyped with MERS-CoV S glycoprotein carrying the D510A mutation on the S protein that inhibits its association with its receptor DPP4 were also generated to be used as a negative control. Expression of MERS CoV S protein in supernatants was confirmed by western blot (Figure 1A left panel). Supernatants collected from transfected cells were used after normalization to HIV-1 p24 protein, to infect THP-1 cells or primary monocytes in subsequent experiments. To confirm the presence of the virus and its infectivity, infected cells were harvested, lysed and luciferase activity was measured in the protein lysates (Figure 1A, right panel). The significantly increased viral entry of the viral particles pseudotyped with MERS-CoV expressing wild-type Spike glycoprotein compared to the D510A mutant Spike protein [17] was successfully confirmed. The pNL4-3.Luc.R-E- pseudotyped with the NL4-3 envelope successfully entered the target cells as previously described [25, 26].

THP1 monocytes expressed DPP4 the levels of which were increased upon differentiation to macrophages by PMA, the ligand of S glycoprotein, while LPS treatment did not affect DPP4 levels in THP1 macrophages (Supplematary Figure S1). THP-1 macrophages, as derived following PMA treatment, were infected with lentiviral particles carrying the wild-type (WT S) or the mutant (D510A) MERS-CoV S protein or were mock-infected. Twenty-four hours post infection, cells were stimulated with LPS and the levels of TNFα and IL-6 were measured. Exposure of THP-1 macrophages to lentiviral particles expressing the MERS CoV S protein resulted in suppression of LPS-induced IL-6 and TNFα production (Figure 1C, 1E). The wild-type MERS-CoV S protein also suppressed basal TNFα and IL-6 production from THP-1 macrophages (Figure 1B, 1D), even though the levels of IL-6 were approaching the detection limit of the assay. We further assessed whether lentiviral particles expressing the MERS-CoV S protein affect macrophage survival and proliferation. We, thus, performed MTT assay in THP1 macrophages infected with letivirus expressing MERS-CoV S glycoprotein for 24, 48 and 72 hours. The results showed that lentiviral particles expressing the MERS-CoV S glycoprotein did not affect macrophage survival and proliferation, even though a small decrease of proliferation was observed in both mutant and WT S glycoprotein-containg particles due to the infection process (Supplementary Figure S2).

### MERS-CoV S glycoprotein induced the production of the immunosuppressive cytokine IL-10

**Figure 2:**
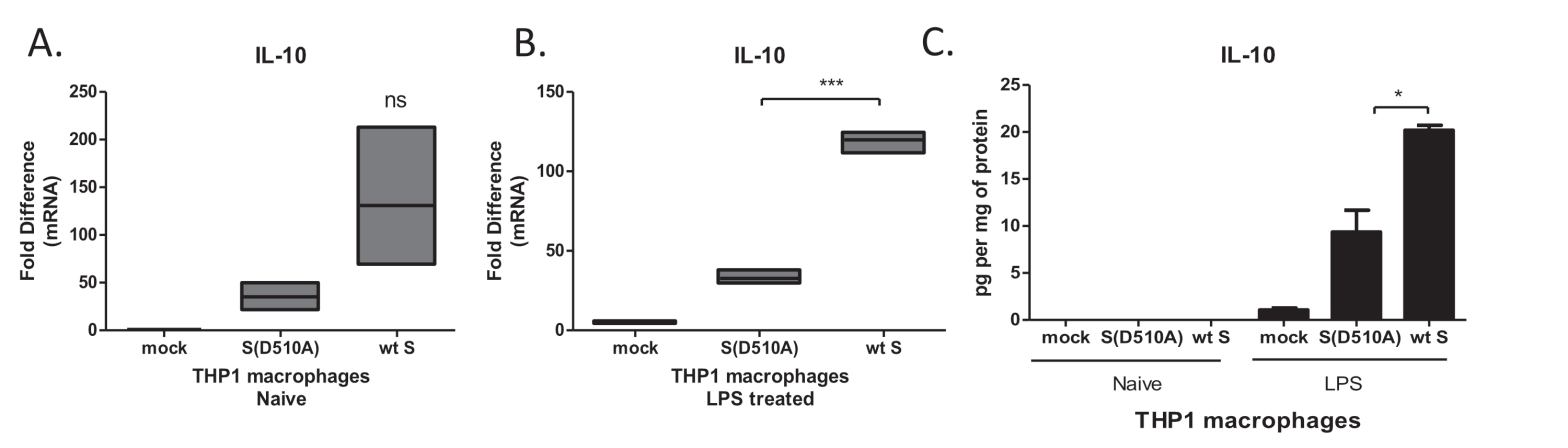
MERS-CoV S glycoprotein augmented LPS-induced IL-10 production **A**., **B**. THP-1 macrophages were infected with lentiviral particles pseudotyped with MERS wild type (wt) or the D510A mutant S glycoprotein (D510A) of MERS-CoV, or left uninfected (mock), and were stimulated with LPS. IL-10 mRNA was measured in the cell extracts. **C**. IL-10 protein was measured in the supernatants of infected THP1 macrophages at the naïve state and following LPS stimulation. ns: not significant, * *p* < 0.05, ***p < 0.001

IL-10 is major immunosuppressive cytokine dampening both innate and adaptive immune responses. Exposure of THP-1 macrophages to particles expressing wild-type MERS-CoV S glycoprotein augmented LPS-induced IL-10 mRNA in THP-1 macrophages (Figure 2A, 2B). Accordingly, infection with the lentiviral particles carrying the wild type S protein augmented LPS-induced IL-10 protein secretion (Figure 2C), supporting increased immunosuppressive capacity of macrophages exposed to MERS-CoV S glycoprotein.

### MERS-CoV S glycoprotein induced expression of the negative regulators of macrophage activation IRAK-M and PPARγ *via* DPP4 receptor

**Figure 3:**
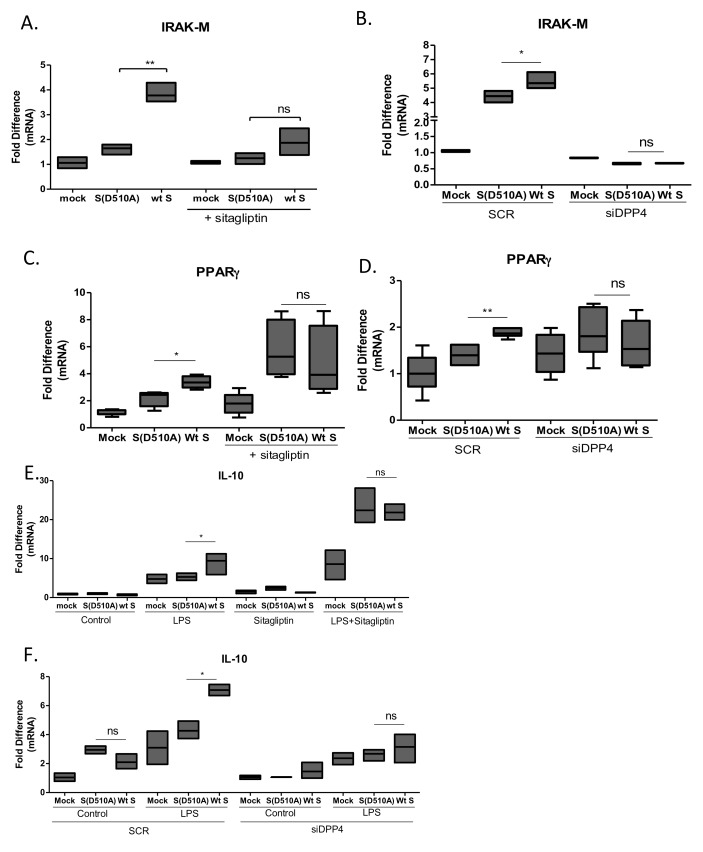
Interaction of MERS-CoV S glycoprotein with DPP4 induced IRAK-M and PPARγ expression **A**., **B**. THP-1 macrophages were infected with lentiviral particles pseudotyped with MERS wild type (wt) or the D510A mutant S glycoprotein (D510A) of MERS-CoV, or left uninfected (mock) in the presence or absence of the DPP4 inhibitor sitagliptin or siRNA targeting DPP4. 24 hours post-infection RNA was isolated and the levels of IRAK-M **A**., **B**. and PPARγ **C**., **D**. and IL-10 **E**., **F**. were measured. ns: not significant, * *p* < 0.05, **p < 0.01

**Figure 4:**
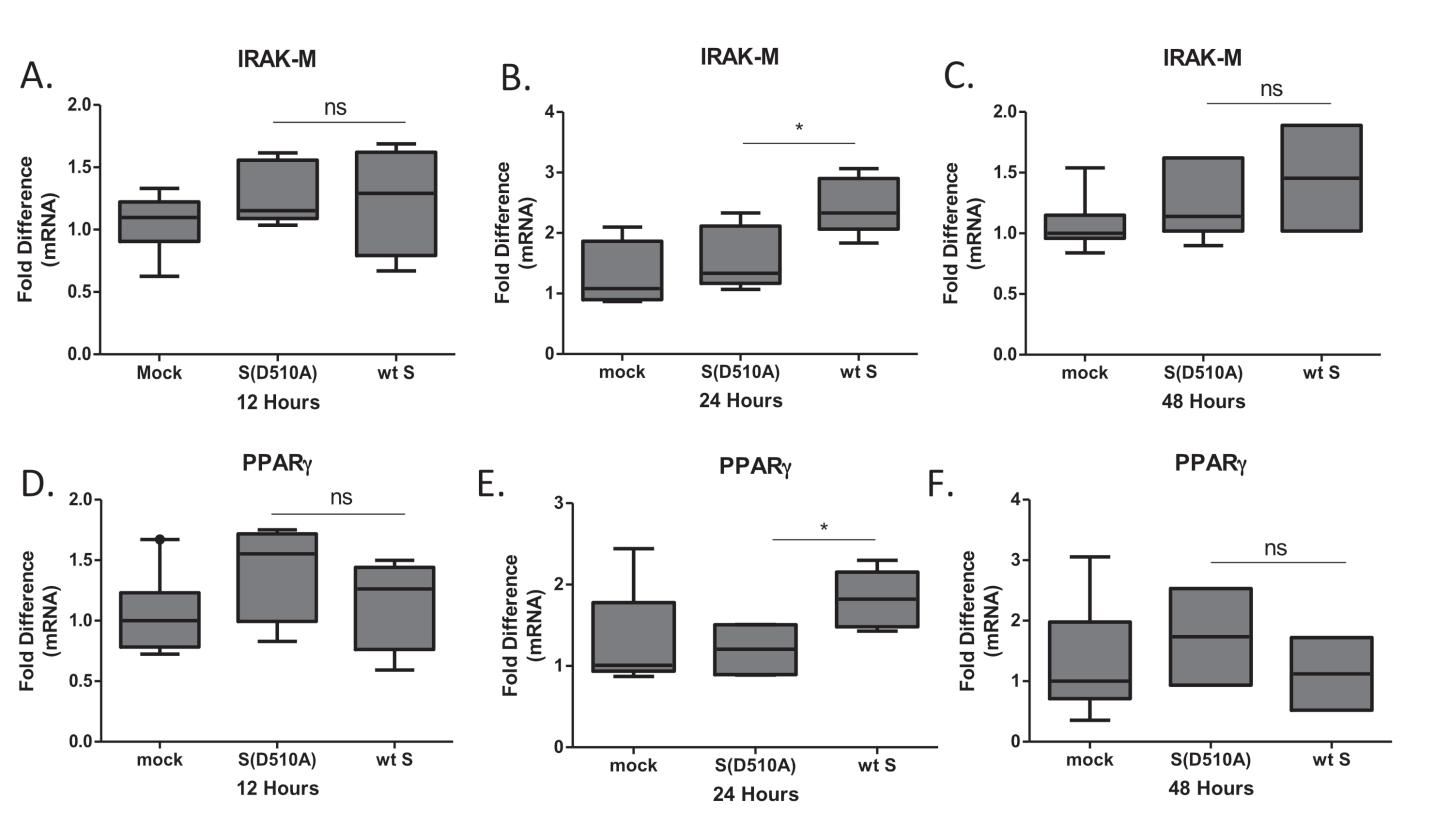
Time-course of IRAK-M and PPARγ expression following infection THP-1 macrophages were infected with lentiviral particles pseudotyped with MERS CoV wild type S glycoprotein (wt) or the D510A mutant S glycoprotein (D510A) of MERS-CoV, or left uninfected (mock) for 12, 24 and 48 hours. Timepoint 0 corresponds to mock-treated cells. RNA was isolated at the respective time-points and the levels of IRAK-M **A**.-**C**. and PPARγ **D**.-**F**. were measured by RT-PCR. Normalization was performed against mock-treated cells in each time point. ns: not significant, * *p* < 0.05.

The magnitude of macrophage activation is regulated by multiple factors including the transcription factor PPARγ, which is known to suppress production of cytokines and IRAK-M, an inactive IRAK kinase isoform known to suppress TLR signaling. Exposure of THP-1 macrophages to lentiviral particles carrying the MERS-CoV S glycoprotein resulted in induction of IRAK-M (Figure 3A) and PPARγ (Figure 3B) expression, suggesting a potential mechanism for its immunosuppressive actions. Exposure of THP1 cells to the same lentiviral particles for different time points revealed that IRAK-M and PPARγ were induced 24 hours following infection and not earlier, suggesting that it was not an immediate-early effect but may affect macrophage responses at later stages of infection (Figure 4). To test whether the amount of S glycoprotein affected IRAK-M induction, we performed serial dilutions of lentiviral particles containing either wild type or D510A mutant S glycoprotein, infected THP1 macrophages and measured IRAK-M. Reducing the amount of Spike at the infection medium failed to induce IRAK-M (Supplementary Figure S3).

MERS-CoV S glycoprotein binds on DPP4 receptor. To determine whether induction of the negative regulators of macrophage activation IRAK-M, PPARγ and IL-10 depended on signaling initiated by the interaction of MERS-CoV S glycoprotein with DPP4, we treated macrophages with the DPP4 enzymatic inhibitor sitagliptin and exposed them to lentiviral particles carrying MERS-CoV wild-type or mutant S glycoprotein. The results confirmed that inhibition of DPP4 signaling ameliorated the induction of IRAK-M (Figure 3A), PPARγ (Figure 3C) and IL-10 (Figure 3E) by the wild-type MERS-CoV S glycoprotein. Interestingly, the DPP4 inhibitor augmented LPS-induced IL-10 expression, as expected because of its anti-inflammatory properties. To further confirm that IRAK-M, PPARγ and IL-10 were induced *via* the interaction of S glycoprotein with DPP4, we used siRNA approach to knock-down DPP4 in THP1 macrophages prior to infection (Supplementary Figure S4). The results showed that knock-down of DPP4 ameliorated the induction of IRAK-M, PPARγ and IL-10 by S glycoprotein (Figure 3B, 3D, 3F).

**Figure 5:**
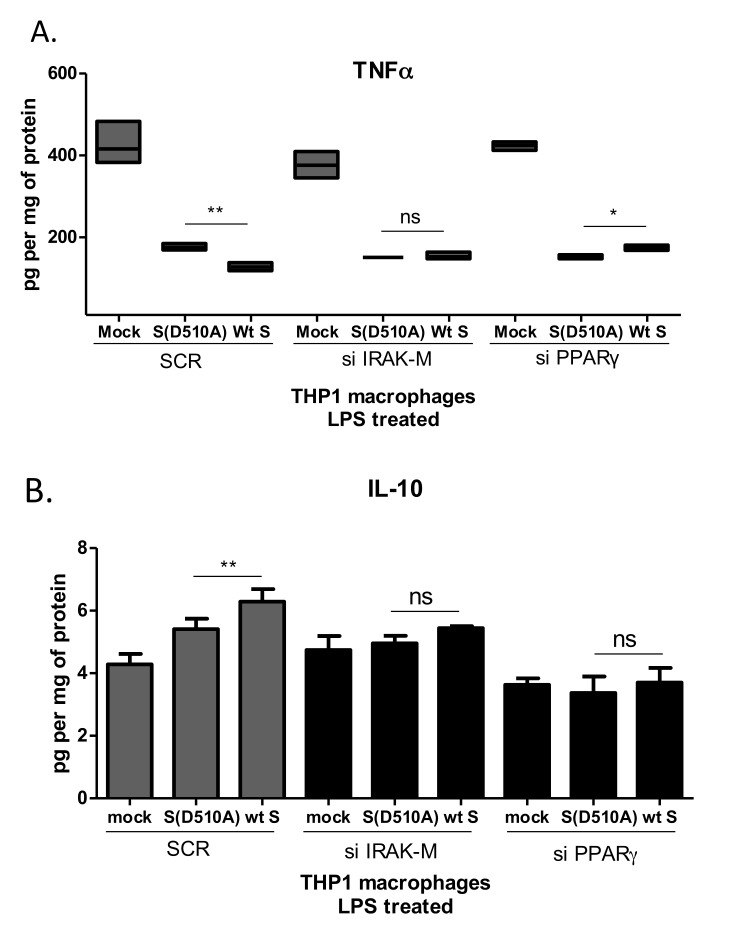
Suppression of TNFα and induction of IL-10 by MERS-CoV S glycoprotein is mediated by IRAK-M and PPARγ THP1 macrophages were transfected with siRNAs targeting IRAK-M or PPARγ and subsequently infected with lentiviral particles pseudotyped with MERS CoV wild type S glycoprotein (wt) or the D510A mutant S glycoprotein (D510A) of MERS-CoV, or left uninfected (mock). Production of TNFα **A**. and IL-10 **B**. in response to LPS was measured in culture supernatants. ns: not significant, * *p* < 0.05, **p < 0.01

Since IRAK-M and PPARγ were both induced by S glycoprotein in macrophages, we decided to check whether the effect of S glycoprotein on TNFα and IL-10 production was mediated by IRAK-M and PPARγ. To test this hypothesis, we transfected THP1 macrophages with siRNAs targeting IRAK-M and PPARγ prior to infection with the lentiviral particles (Supplementary Figure S4). Following knock-down, cells were infected with lentiviral particles containing either wild type S or D510A mutant S glycoprotein or were mock infected prior to stimulation with LPS. The results showed that knock-down of IRAK-M and PPARγ reversed the effect of WT S glycoprotein on TNFα (Figure 5A) and IL-10 (Figure 5B). Suppression of PPARγ augmented LPS-induced TNFα production in cells infected with letiviral particles carrying WT S glycoprotein, supporting the importance of PPARγ as a negative regulator of TNFα expression.

### MERS-CoV S glycoprotein suppressed TNFα, induced IL-10, IRAK-M and PPARγ expression in primary human monocytes

**Figure 6:**
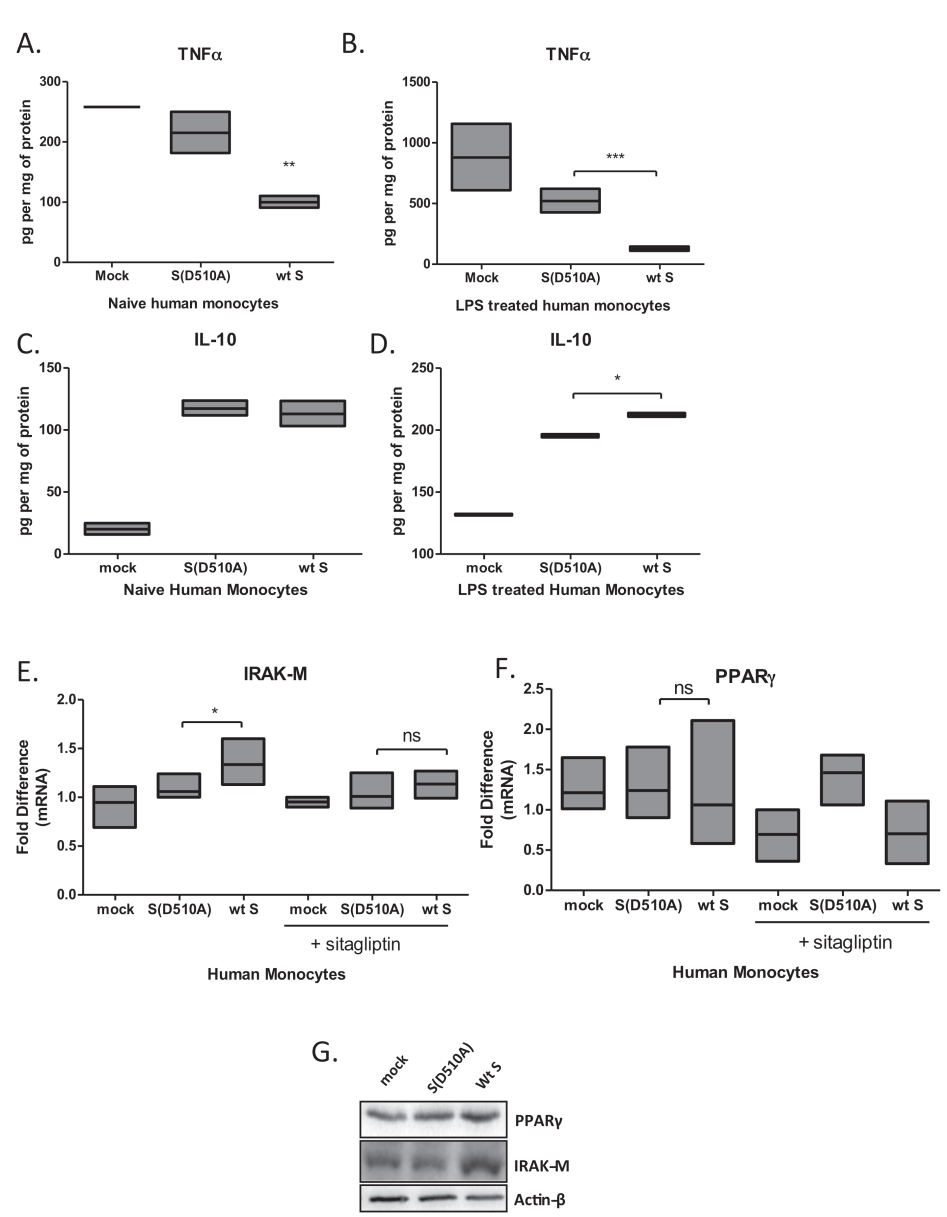
Infection of primary human monocytes with MERS-CoV S glycoprotein pseudotyped particles Primary human monocytes were isolated from buffy coats of healthy human donors and were infected with lentiviral particles pseudotyped with MERS wild type (wt S) or the D510A mutant S glycoprotein (D510A) of MERS-CoV, or left uninfected (mock) for 24 hours, in the presence or absence of the DPP4 inhibitor sitagliptin, and were stimulated with LPS. TNFa **A**.,**B**. and IL-10 **C**.,**D**. secretion was quantified by ELISA. Expression of IRAK-M **E**. and PPARγ **F**. mRNA and protein **G**. in naïve human macrophages quantified by RT-PCR and protein levels by Western blot, respectively. Results are representative of three independent experiments. ns: not significant, * *p* < 0.05, **p < 0.01, ***p < 0.001

To confirm our findings in primary human cells, we isolated primary human monocytes from healthy donors and infected them with lentiviral particles carrying MERS-CoV wild-type or mutant S glycoprotein. PBMCs expressed high levels of DPP4 (Supplementary Figure S1), the receptor for MERS CoV S glycoprotein. The results confirmed the findings in THP1 cells showing that lentiviral particles carrying MERS-CoV wild-type S protein suppressed basal and LPS-induced TNFα production (Figure 6A, 6B) and augmented LPS-induced IL-10 secretion (Figure 6C, 6D). In addition, lentiviral particles carrying MERS-CoV wild-type S protein induced expression of IRAK-M mRNA and protein (Figure 6E, 6G) while PPARγ was only induced at the protein level (Figure 6F, 6G). Induction of IRAK-M and PPARγ was ameliorated in the presence of the DPP4 inhibitor sitagliptin (Figure 6E-6F).

## DISCUSSION

MERS-CoV infection is associated with severe ARDS and increased mortality of infected individuals. It infects several cell types including immune cells, utilizing DPP4/CD26 as receptor, a receptor present in most immune cells including macrophages. MERS-CoV infection alters both innate and adaptive immune responses. In the present study, we demonstrated that the DPP4-binding protein of MERS-CoV, the S glycoprotein, altered macrophage responses rendering them hypo-responsive to TLR4 stimulation. This finding indicates that interaction of MERS-CoV S protein with DPP4 initiates signals that suppress macrophage activation supporting an immunomodulatory mechanism of MERS-CoV that may allow viral replication and expansion.

Recent evidence have shown that infection of human T-cells with MERS-CoV results in suppression of immune responses by inducing both intrinsic and extrinsic apoptosis pathways [19], providing a potential mechanism for the enhanced pathogenicity of the virus. The same group has shown that macrophages can also be infected by MERS-CoV and in this case infection with the virus induces the production of TNFα, IL-6, IFNγ and IL-12, thus initiating anti-viral responses [24] and additional evidence showed increased IFNλ, and several chemokines such as CXCL10, CCL2, CCL3 and IL-8 production from macrophages or dendritic cells, in the absence [27, 28] or presence [24, 29] of viral replication. Our findings showed that the MERS-CoV S protein did not induce the production of TNFα and IL-6 but was rather capable of suppressing their induction by LPS. This suggests that the induction of these factors observed in earlier studies was due to the active replication of the virus observed, since in that case macrophages were infected with an active virus [24]. Indeed, the induction of the negative regulators of macrophage activation IRAK-M and PPARγ occurred 24 hours following exposure to MERS-CoV S protein, suggesting that the virus may exert a pro-inflammatory action at the early stages of infection followed by a state of reduced responsiveness to pro-inflammatory stimuli. This effect was also observed in primary human monocytes, where IRAK-M was induced at both mRNA and protein levels while PPARγ induction was observed only at the protein level, potentially due to the heterogeneity of PBMCs, which may mask the effect.

Earlier evidence has shown that lack of PPARγ resulted in increased lethality in mice infected with Influenza virus [30], and H3N2 ifluenza virus infection PPARγ was induced *via* Fatty Acid Binding Protein 5 (FAPB5) to suppress immune responses [31], supporting the crosstalk of PPARγ with viral infection and innate immune responses. A PPARγ polymorphism (Pro12Ala) has been associated with sustained response to Hepatitis C Virus [32]. IRAK-M levels in peripheral monocytes and macrophages regulate inflammatory responses in humans [33] and in alveolar macrophages [34] but the effect of viral infection on IRAK-M expression has not been previously demonstrated. Analysis of genetic variants of IRAK-M has only been performed in association with Systemic Lupus Erythematosus, but no association was found [35]. Thus, our results provide evidence for the crosstalk of IRAK-M and viral infection and support earlier findings on the effect of influenza viruses on PPARγ expression and function.

Our findings support that MERS-CoV S glycoprotein did not mediate the pro-inflammatory effects observed during active viral replication. In addition, expression of IFNγ was not induced in THP-1 macrophages infected with a lentiviral particles carrying the S glycoprotein (data not shown), further supporting that interaction of the MERS-CoV S glycoprotein with DPP4 and initiation of DPP4-dependent signals did not trigger anti-viral responses. Our findings demonstrate suppression of TNFα production not only upon LPS stimulation but also at basal levels in THP1 macrophages and primary human monocytes, supporting that MERS-CoV S glycoprotein induced a mechanism that affects not only TLR signals but also alternative pathways that control cytokine production. MERS-CoV S glycoprotein also induced the expression of IL-10 from LPS-activated macrophages, supporting an additional mechanism for suppressing immune responses. Thus, our findings support that signaling initiated by MERS-CoV S protein through DPP4 renders macrophages hypo-responsive to activation signals, promoting immune suppression in cells where the virus does not actively proliferate. As a result, suppression of macrophage responses allows viral propagation and suppresses the capacity of the host to respond to the same or other opportunistic infections, thus contributing to the increased mortality of MERS-CoV infected patients.

Macrophage activation is regulated at different levels and factors. IRAK-M is an inactive homolog of IRAK kinases and is known to suppress macrophage activation by TLRs thus rendering them hypo-responsive to pro-inflammatory triggers. Expression levels of IRAK-M highlight their responsiveness and are regulated by various factors including lung surfactants as well as TLR ligands and adipokines [33, 34, 36]. In the present report, we demonstrated that the S glycoprotein of MERS CoV induced the expression of IRAK-M in macrophages, providing a potential molecular mechanism of the suppression of cytokine production. Induction of IRAK-M occurred 24 hours following infection suggesting that MERS S glycoprotein only affected late responses of macrophages, which may provide evidence for immunosuppression observed at later stages of infection.

Responsiveness of macrophages is also regulated by the transcription factor PPARγ, a transcriptional repressor that can suppress transcription of pro-inflammatory cytokines such as TNFα and IL-6 [37]. Our results showed that MERS-CoV S glycoprotein induced the expression of PPARγ providing an additional molecular mechanism for its immunosuppressive action. Given the fact that PPARγ is expressed not only in macrophages but also in T-cells and epithelial cells, this mechanism may not be restricted to macrophages.

The S glycoprotein of MERS-CoV binds on DPP4 receptor allowing its entry into the host cell. Our studies showed that this interaction does not only facilitate viral entry but it also initiated signals that mediated immunosuppressive action to allow the virus to propagate itself. This immunosuppression may account for increased pathogenicity of the virus.

Our findings demonstrated that indeed the immunosuppressive action of the S glycoprotein is mediated by DPP4 since inhibition of DPP4 by sitagliptin, a DPP4 inhibitor, ameliorated induction of IRAK-M and PPARγ. Sitagliptin and other DPP4 inhibitors are known to act as anti-diabetic agents reversing insulin resistance. It is yet unknown whether individuals that are under treatment with sitagliptin are less susceptible to MERS-CoV infection or whether they do not produce as severe symptoms of the disease. Moreover, our findings may propose the use of sitagliptin as a potential treatment of patients with MERS-CoV infection since it may reverse the immunosuppressive actions of the virus.

**Figure 7:**
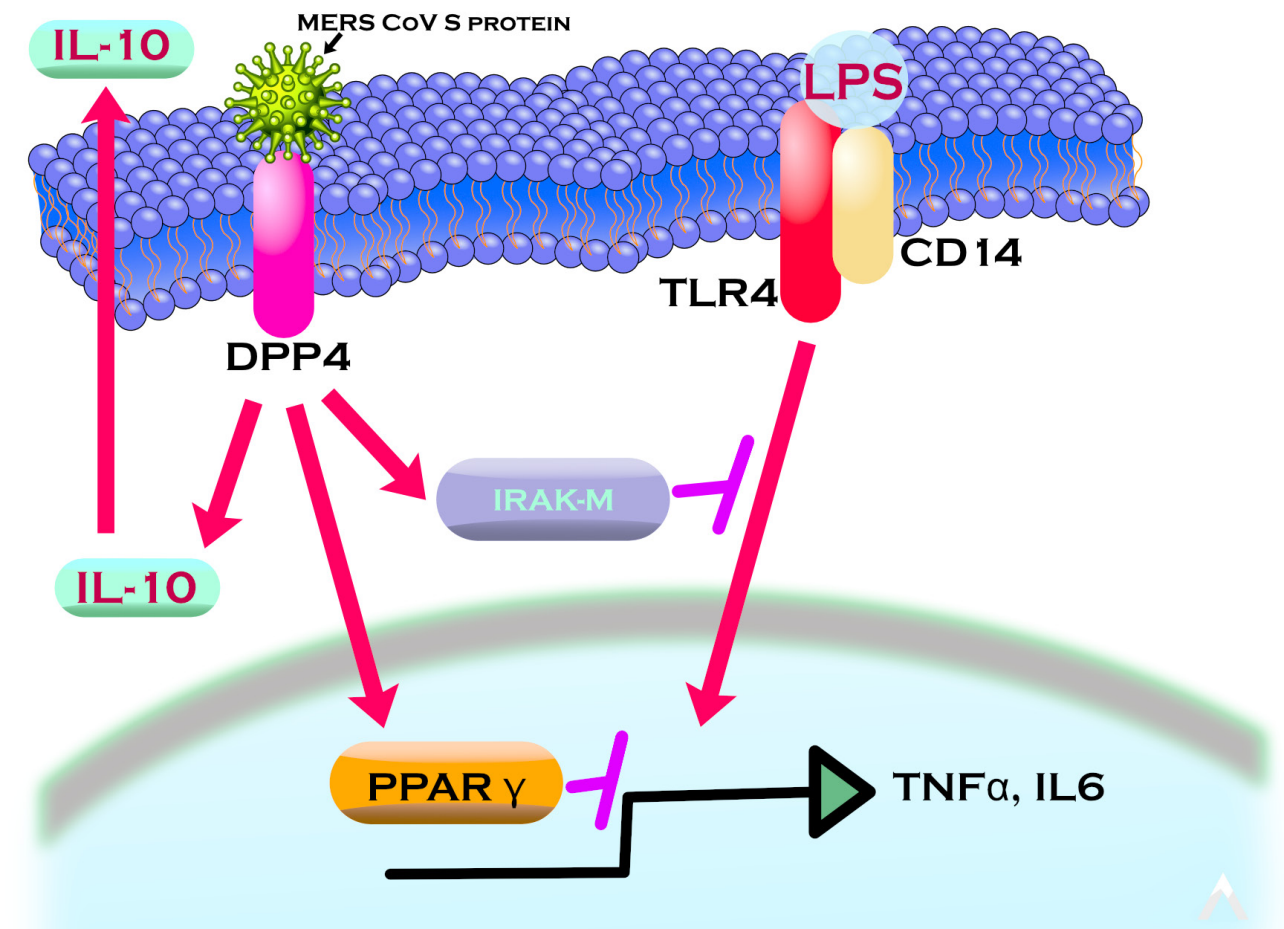
Model of MERS-CoV S glycoprotein immunosuppressive action

The present report demonstrates a novel mechanism for MERS-CoV S protein action, being the suppression of macrophage responses *via* induction of IRAK-M, PPARγ and the immunosuppressive cytokine IL-10 (Figure 7). This effect was reversed by the DPP4 inhibitor sitagliptin, highlighting the role of S glycoprotein/DPP4 interaction in the pathogenicity of the virus and providing a potential means of reversing the pathogenicity of MERS-CoV infection.

## MATERIALS AND METHODS

### Cells

THP-1 monocytic cell line was maintained in suspension in RPMI 1640 medium, supplemented with 1% L-glutamine, 1% sodium pyruvate, 50 μM β-mercaptoethanol, 10% fetal bovine serum (FBS) and antibiotics (10,000 U/ml penicillin and 10,000 μg/ml Streptomycin) [33]. For differentiation experiments, THP-1 cells were seeded at a final density of 1×10^6^ cells/ml in 6-well plates. Cell differentiation was induced by 10 ng/ml phorbol 12-myristate 13-acetate (PMA) (Sigma-Aldrich) to the medium. Differentiated (adherent) THP-1 macrophages were used for infection experiments. Peripheral Blood Mononuclear Cells (PBMCs) were isolated from buffy coats obtained from human peripheral blood of healthy donors by Ficoll density gradient centrifugation. Monocytes were selected by adherence to plastic in RPMI for 2 hours.

### Generation of lentiviral particles pseudotyped with MERS S glycoprotein

Production of lentiviral particles pseudotyped with MERS S glycoprotein was performed as previously described [17]. Briefly, the human immunodeficiency virus backbone expressing firefly luciferase (pNL4.3R-E-luciferase) were co-transfected into 293T cells, either with the MERS-CoV wild-type spike glycoprotein (Wt S) expression vector (pcDNA3.1+; Invitrogen) or with the mutant D510A spike glycoprotein vector which significantly reduces its binding with DPP4 and consequently the viral entry [17] (Figure 1A). Supernatants containing the lentiviral particles pseudotyped with MERS-CoV S glycoprotein were harvested 48 h later and were subsequently normalized using a p24 ELISA kit prior to infection of the target cells (Lenti-X™ p24 Rapid Titer Kit, Takara Bio USA, Inc.). The amount of Spike glycoprotein incorporated in the different types of the generated viral particles pseudotyped with the MERS-CoV S protein was determined by semi-quantitative Western blot in purified viral particles, ensuring an equal MERS-CoV S glycoprotein expression among them. Infected cells were lysed at 48 h after infection and viral entry efficiency was quantified by comparing the luciferase activity between the pseudotyped viruses either not expressing the MERS-CoV S glycoprotein or bearing the mutant and wild-type MERS-CoV S glycoprotein.

### Transfection

Transient transfection was performed using the Lipofectamine Reagent (RNAiMAX, Life Technologies) as per manufacturer's instructions. In short, THP1 cells with PMA added were seeded at approximately 80% confluency. The next day silencing RNA molecules (siRNA) were used at a final concentration of 20nM to transfect the cells. Cells were cultured for 24 hours in the presence of the transfection reagents prior to infection and LPS treatment, the following, pre-validated siRNAs used were: IRAK-M siRNA (sc-39098, Santa-Cruz), PPARγ siRNA (sc-29455, Santa-Cruz), CD26 siRNA (sc-42762, Santa-Cruz) and Silencer Negative Control (SCR) siRNA (AM4611g, Ambion).

### ELISA

For the detection of the levels of IL-6, TNFα and IL-10 that were produced from cultured THP-1 cells Enzyme Linked ImmunoSorbent Assay (eBioscience; detection limit 2pg/ml for all cytokines) was performed as indicated by the manufacturer and as previously described [38].

### MTT

Cells were seeded at ∼80% confluency in a 96-well plate for the representative time-points, 4 hours prior to the endpoint 11 μl of MTT reagent was added per 100 μl of cell culture medium. At endpoints, medium was removed from wells and 100 μl isopropanol/HCl was added to the cells. Cells were incubated for 5 minutes in a shaker incubator and then OD at 595nm was measured.

### Western blot

For Western blot, cell lysates cells were harvested using the M-PER Mammalian Protein Extraction Reagent (Thermo Scientific, Rockford, IL, USA), enriched with the Halt-Protease Inhibitor Cocktail, EDTA-free (PIERCE, Rockford, IL, USA) according to the manufacturer's instructions and as previously described [39, 40]. Proteins were separated on 10% polyacrylamide gels containing Sodium-Dodecyl Sulphate, and then transferred to nitrocellulose membranes. The MERS-CoV S glycoprotein protein as expressed by the pcDNA3.1+ vector is tagged with C9 tag (TETSQVAPA) and thus the anti-C9 antibody [aE11] (ab90801, Abnova) was used for the detection of the protein. In the context of pseudoviral particles pseudotyped with MERS-CoV expressing Spike glycoportein, the migration pattern of the S viral protein presents two major bands; a high molecular weight band of approximately 190 KDa and a low molecular weight band of 80 KDa, accompanied by a faint band of ∼100 KDa as previously described [41]. For the detection of PPARg and IRAK-M, primary antibodies (anti-PPARγ (E-8):sc-7223, Santa Cruz Biotechnology and anti-IRAK-M (ab-8116-Abcam) were incubated with membrane overnight at 4°C, washed with PBST (0.1%Tween) and then incubated with HRP-conjugated secondary antibody for 1 hour at room temperature. Visualization of membranes was performed using the ECL system (Pierce) and a ChemiDoc^TM^ XRS+ (Bio-Rad). Band intensity for protein quantification was measured using ImageLab^©^ Software (Bio-Rad).

### Real-time PCR

For the detection of mRNA levels of IRAK-M, PPARg and IL-10, total RNA was extracted from macrophages using TRI Reagent (SIGMA Life Science) as previously described [38]. Briefly, 3×10^5^ cells were pelleted, washed with PBS, resuspended in 250 μl of TRI Reagent and 50 μl of chlorophorm were added. The samples were centrifuged at 12,000 x g at 4°C for 15 min. After centrifuging the aqueous phase was removed and transferred to a new tube in which 125 μl of 100% isopropanol were added to precipitate RNA. Following washing with 75% ethanol pellet was dried and resuspended in 20μl of RNase- free water. One microgram of total DNAse-digested RNA was used for cDNA synthesis (Thermoscript RT; Invitrogen, Carlsbad, CA). The SYBR Green method was followed in the PCR reaction. Primer sequences were the following: IRAK-M: 5′CACAACGTTCAACCATGCTC3′and 5′ TGTTTACTGCTGCTGCTGGT3′; PPARγ: 5′ GCTGGCCTCCTTGATGAATA3′ and 5′ TTGGGCTCCATAAAGTCACC3′; DPP4: 5′ GGTTCTGCTGAACAAAGGCA 3′ and 5′ TCTCCAAGAAAACTGAGCTGT 3′. Actin-b was used as a reference gene: 5′ GCCGTGCTGTCCCTCTAC 3′ and 5′ AGCGCG TAGCCCTCATAAAT - 3′. Denaturation was carried out at 95°C for 20 seconds, annealing at 60°C for 15 seconds and extension at 72°C for 30 seconds, for 40 cycles.

### Statistical analysis

Statistical analysis was performed using GraphPad Prism version 5.00 for Windows, GraphPad Software, San Diego California USA, www.graphpad.com and *p* value < 0.05 was considered as indicative of statistical significance. One-way ANOVA and *t*-test analyses were performed.

## SUPPLEMENTARY FIGURES



## Abbreviations

MERS CoV: Middle East Respiratory Syndrome Corona Virus
DPP4: Dipeptidyl-Peptidase 4
TNFα: Tumor Necrosis Factor α
IL-(6): Interleukin-(6)
IRAK-M: Interleukin-1 Receptor Associated Kinase -M
PPARγ: Peroxisome Proliferator-Activated Receptor γ
TLR: Toll Like Receptor
LPS: Lipopolysaccharide
PMA: phorbol 12-myristate 13-acetate
PBMC: Peripheral Blood Mononuclear Cells
ARDS: Acute Respiratory Distress Syndrome
IFN: Interferon
CXCL: Chemokine (C-X-C motif) ligand
CCL: Chemokine (C-C motif) ligand
