# Supplementary material for: Middle east respiratory syndrome corona virus spike glycoprotein suppresses macrophage responses via DPP4-mediated induction of IRAK-M and PPARγ
Source: Oncotarget. 2017 Jan 19;8(6):9053–66. doi: 10.18632/oncotarget.14754 (PMC5354714; doi:10.18632/oncotarget.14754)
Supplement: Supplementary file 1 [file oncotarget-08-9053-s001.pdf]

Middle east respiratory syndrome corona virus spike glycoprotein suppresses macrophage responses *via* DPP4-mediated induction of IRAK-M and PPARγ

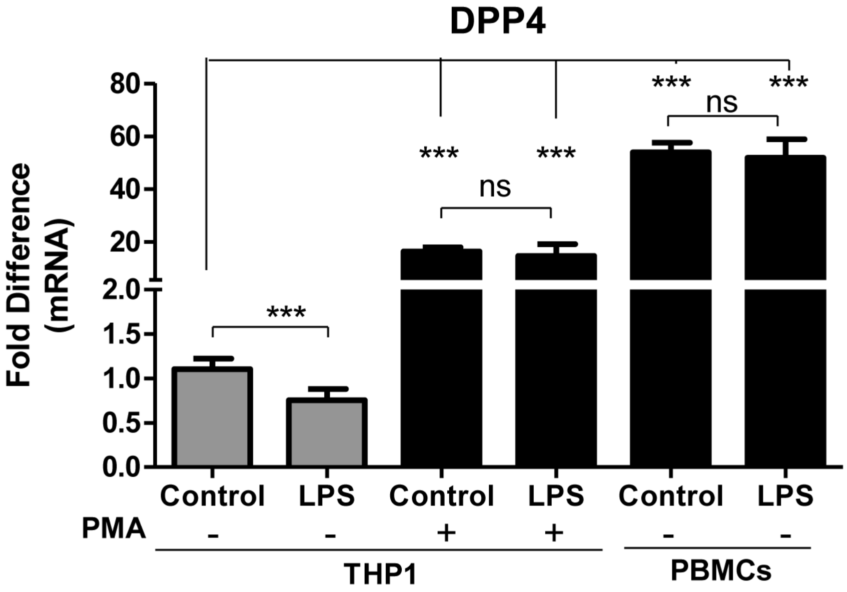

Figure S1: DPP4 expression in THP1 cells and PBMCs. THP1 cells were cultured in the presence or absence of PMA and LPS and RNA expression of DPP4 was analyzed. THP1 monocytes expressed basal levels of DPP4. Upon PMA treatment DPP4 expression was induced. Peripheral Blood Mononuclear Cells (PBMCs) also expressed high levels of DPP4. LPS did not alter DPP4 mRNA levels in macrophages (PMA treated THP1 and PBMCs). In contrast, THP1 monocytes showed decreased expression after LPS treatment. Data are expressed as mean +/- SD. ***p<0.001, ns: not significant.

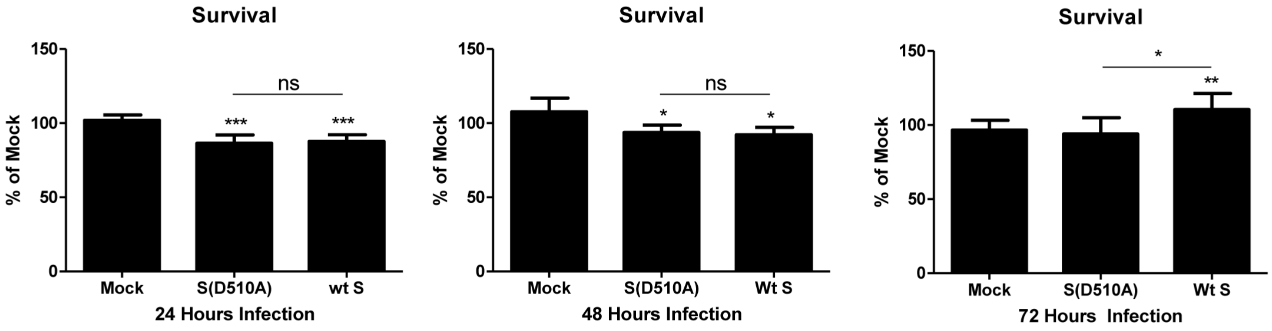

Figure S2: Survival of infected cells. THP1 macrophages were infected with either wt Spike (Wt S), D510A mutant Spike (S(D510A)) or were mock infected for the respected time-points and survival of cells was measured by the MTT assay. All samples were normalized to mock infected cells. Data are expressed as mean +/- SD. *p<0.05, **p<0.01, ***p<0.001, ns: not significant.

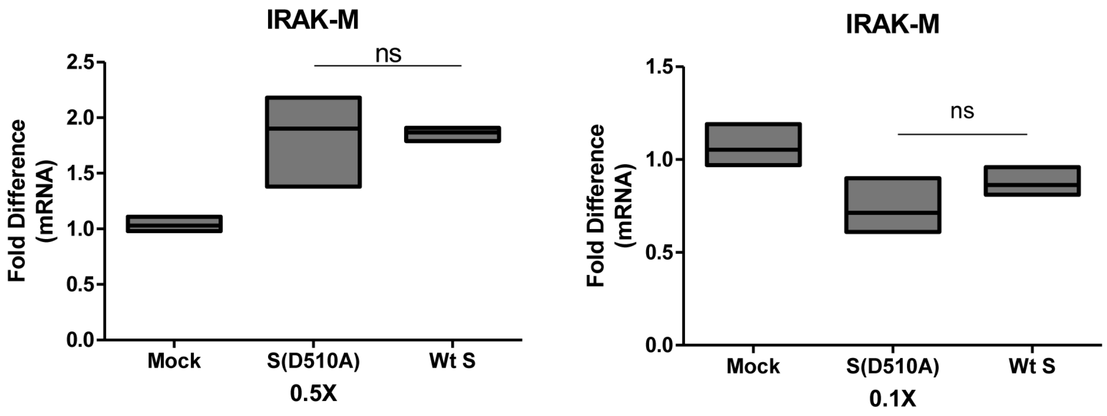

Figure S3: Effect of the amount of S glycoprotein on IRAK-M expression. THP1 macrophages were infected with dilutions of medium containing lentiviral particles pseudotyped with MERS-CoV S glycoprotein or D510A mutant S glycoprotein, or were mock infected. Smaller amounts of Spike failed to induce expression of IRAK-M. Data are expressed as mean +/- SD. ns: not significant.

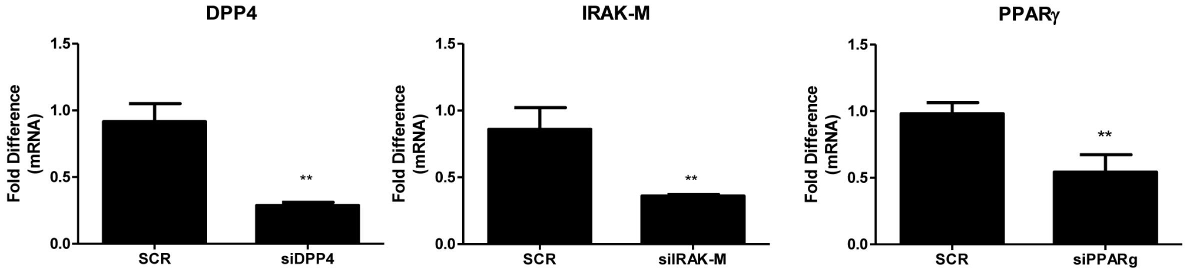

Figure S4: Knock-down of DPP4, IRAK-M and PPARγ after transfection with the respected siRNAs. THP1 macrophages were transfected with siRNAs targeting either DPP4, IRAK-M or PPARγ. 24 hours after transfection RNA was extracted and mRNA levels of DPP4, IRAK-M and PPARγ respectively were measured by RT-PCR. Results showed successful knock-down of each target by the respective siRNA sequence. Data are expressed as mean +/- SD. **p<0.01.
